# Uræmic Amaurosis

**Published:** 1884-09

**Authors:** 


### Editorial.

### Uræmic Amaurosis.

Uræmic amaurosis, a loss of vision more or less completely seen in cases of uræmic "intoxication," but not showing any changes in the fundus of the eye recognizable by the ophthalmoscope, is a subject that receives but a passing notice in the text-books on ophthalmology.

Dr. A. Friedenwald, in the *Medical News* for August 8, 1884, says that transient loss of vision is observed among other symptoms of uræmic intoxication, but that pathology does not satisfactorily explain just how the eye becomes involved. It may occur in cases where it would be least expected, and, on the other hand, it may be absent in cases in which uræmic symptoms are most exaggerated. In some cases it is the only prominent symptom. The same irregularity is observed in the appearance of amaurosis as in that of other symptoms of uræmia. We find it equally difficult to explain the absence of all uræmic symptoms in many cases of kidney disease, in which the urinary analysis and the very pronounced dropsy testify to the existence of grave lesions; while, on the other hand, we are sometimes suddenly confronted with very threatening uræmic manifestations, when the urine may show but a trace of albumen.

This anomaly of vision, like all other signs of uræmia, is characterized by the suddenness of its invasion, the loss of vision from the first, or in a very short time, being complete. The ophthalmoscopic examination will yield entirely negative results, except where retinal degeneration pre-existed. The amaurosis is not restricted to any special form of kidney disease, but occurs in all forms, although, perhaps, more frequently in those met with in pregnancy and scarlet fever. Other uræmic symptoms may precede the amaurosis, such as headache and vomiting, and the patient may arouse from a convulsion or a state of coma with sight gone. Or, on the other hand, the loss of sight may be the first and most prominent symptom of uræmic "explosion."

The prognosis, so far as the sight is concerned, is favorable, if the patient does not succumb. It may be re-established as suddenly as it disappeared, or it may return gradually, several days elapsing before full vision is regained. Sometimes vision remains somewhat impaired, permanently. In some cases there are several attacks of blindness.

The diagnosis is to be made between albuminuric retinitis and amaurosis without any apparent changes in the retina or optic nerve, dependent upon uræmia.

Treatment consists in free diaphoresis and purgation, the use of jaborandi and other means of combatting kidney disease, and in pregnancy in inducing premature labor in certain cases with severe uræmic symptoms.

The author concludes:

"1. That when amaurosis suddenly overwhelms a patient in both eyes with no ophthalmoscopic change, uræmia should be suspected, even in the absence of any other prominent uræmia symptom.

"2. That uræmic amaurosis will continue only as long as the uræmia exists, and will disappear when the function of the kidney is re-established. When permanent injury to sight is observed, it may be due to pre-existing retinal changes, not at all uncommon in Bright's disease.

"3. That the chances for a full return of sight are somewhat impaired when the patient has been the subject of recurring attacks.

"4. That by exhibiting jaborandi and other means for inducing free diaphoresis, and by free purgation, a catastrophe may be averted in the general forms of uræmia; but when it occurs in pregnancy, premature labor is the only remedy which promises safety to the patient."

